# Triaqua­bis­{2-meth­oxy-6-[(phenyl­iminium­yl)meth­yl]phenolate-κ*O* ^1^}manganese(II) dinitrate

**DOI:** 10.1107/S1600536811019593

**Published:** 2011-05-28

**Authors:** Guo-Di Ge, Jin-Bei Shen, Guo-Liang Zhao

**Affiliations:** a College of Chemistry and Life Science, Zhejiang Normal University, Jinhua 321004, Zhejiang, People’s Republic of China; b Zhejiang Normal University Xingzhi College, Jinhua, Zhejiang 321004, People’s Republic of China

## Abstract

The crystal structure of the title compound, [Mn(C_14_H_13_NO_2_)_2_(H_2_O)_3_](NO_3_)_2_, is comprised of two Schiff base 2-meth­oxy-6-(*N*-phenyl­carboximido­yl)phenol (H*L*) ligands and three coordinated water mol­ecules. The Mn^II^ ion lies on a twofold axis that bis­ects one water O atom. The coordination sphere of the five-coordinate Mn atom is completed by the two monodentate H*L* ligands and three coordinated water mol­ecules binding through their O atoms, affording a distorted tetra­gonal–pyramidal geometry. In the phenolate ligands, the hy­droxy H atom transfers to the imine N atom. This H atom is also involved in an intra­molecular N—H⋯O hydrogen bond that imposes a nearly planar conformation on each ligand, with dihedral angles of 2.78 (3) and 2.43 (5)° between the aromatic rings of each ligand. In the crystal, mol­ecules are linked by inter­molecular O—H⋯O hydrogen bonds.

## Related literature

For Schiff base ligands derived from *o*-vanillin and aniline and their rare earth complexes, see: Garnovskii *et al.* (1993 ▶); Shen *et al.* (2011 ▶); Zhao *et al.* (2006 ▶). For the synthesis of related Schiff bases, see: Burrows & Bailar (1966 ▶).
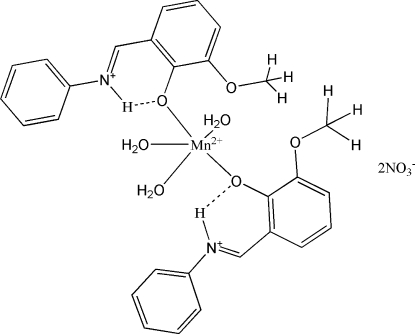


## Experimental

### 

#### Crystal data

[Mn(C_14_H_13_NO_2_)_2_(H_2_O)_3_](NO_3_)_2_
*M* *_r_* = 687.52
Orthorhombic, 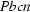

*a* = 17.4364 (3) Å
*b* = 10.4199 (2) Å
*c* = 16.9014 (3) Å
*V* = 3070.74 (10) Å^3^
*Z* = 4
Mo *K*α radiation
μ = 0.50 mm^−1^
*T* = 296 K
0.26 × 0.14 × 0.06 mm

#### Data collection

Bruker APEXII area-detector diffractometer
Absorption correction: multi-scan (*SADABS*; Sheldrick, 1996 ▶) *T*_min_ = 0.918, *T*_max_ = 0.969
11534 measured reflections
2705 independent reflections
1864 reflections with *I* > 2σ(*I*)
*R*_int_ = 0.035

#### Refinement

*R*[*F* ^2^ > 2σ(*F* ^2^)] = 0.043
*wR*(*F* ^2^) = 0.118
*S* = 1.07
2705 reflections
210 parameters
4 restraints
H-atom parameters constrained
Δρ_max_ = 0.22 e Å^−3^
Δρ_min_ = −0.33 e Å^−3^

### 

Data collection: *APEX2* (Bruker, 2006 ▶); cell refinement: *SAINT* (Bruker, 2006 ▶); data reduction: *SAINT*; program(s) used to solve structure: *SHELXS97* (Sheldrick, 2008 ▶); program(s) used to refine structure: *SHELXL97* (Sheldrick, 2008 ▶); molecular graphics: *SHELXTL* (Sheldrick, 2008 ▶); software used to prepare material for publication: *SHELXL97*.

## Supplementary Material

Crystal structure: contains datablocks global. DOI: 10.1107/S1600536811019593/rn2084sup1.cif

Additional supplementary materials:  crystallographic information; 3D view; checkCIF report

## Figures and Tables

**Table 1:** Hydrogen-bond geometry (Å, °)

*D*—H⋯*A*	*D*—H	H⋯*A*	*D*⋯*A*	*D*—H⋯*A*
O1*W*—H1*WA*⋯O5^i^	0.85	2.18	3.032 (3)	180
O1*W*—H1*WB*⋯O4^ii^	0.85	1.96	2.809 (3)	180
O2*W*—H2*WA*⋯O3^ii^	0.85	1.96	2.800 (3)	169
N1—H1*A*⋯O1	0.86	1.92	2.611 (2)	137

Symmetry codes: (i) 
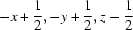
; (ii) 
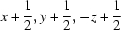
.

**Fig. 1.**
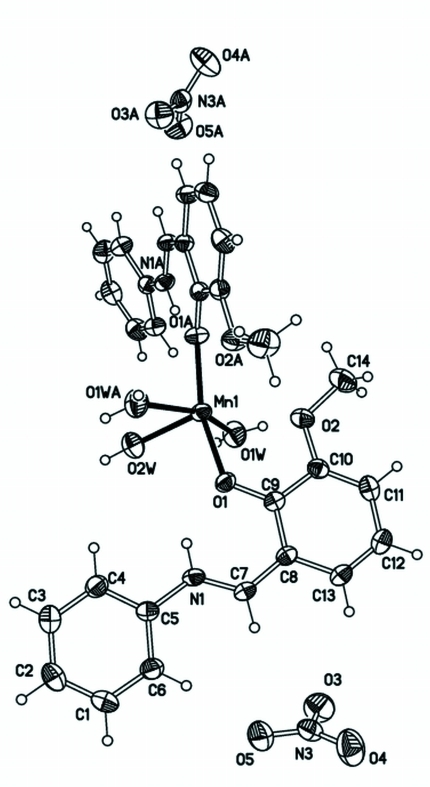
The molecular structure of the title complex, showing the atom-labelling scheme. Displacement ellipsoids are drawn at the 30% probability level. (The atoms labelled with the suffix A are related by the symmetry operation -x + 1,-y,-z + 1)

**Fig. 2.**
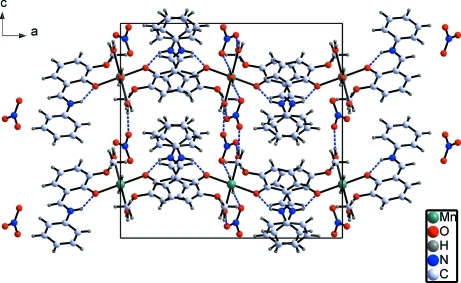
The packing plot of the title compound, showing H-bond interactions (dashed lines).

## supplementary crystallographic information

### Comment

It has well been documented that Schiff bases are important in diverse fields of chemistry and biochemistry owing to their biological activities (Garnovskii *et al.*, 1993). The complexes prepared by ligands derived from *o* -vanillin have attracted considerable attention for a number of years due to the intriguing biological activities of *o*-vanillin and the convenience of the synthesis of related Schiff bases (Burrows & Bailar, 1966). For these reasons, we have been engaged in the syntheses of new Schiff bases derived from *o*-vanillin and their transition and rare earth metal complexes (Shen *et al.* 2011; Zhao *et al.* 2006). Herein, we describe a new Mn^II^ complex.

The structure of complex (1) is shown in Fig. 1, which contains two 2-methoxy-6-(*N*-phenylcarboximidoyl)phenol (H*L*) ligands, three coordinated water molecules and two independent nitrate ions. The coordination sphere of the five-coordinate Mn atom is completed by the two monodentate H*L* ligands and three coordinated water molecules binding through their O atoms, affording a distorted tetragonal pyramid geometry. The coordination geometry around Mn^II^ is better described as a distorted square pyramid with the basal positions occupied by the four O atoms; O1, O1A, O1W and O1WA. The apical position is occupied by O2W. The Mn^II^ ion lies on a twofold axis that bisects O2W. The five Mn—O bond distances are listed in Table 1.The Mn—O (phenolic) bonds are 2.118 (4) Å, which are shorter than the similar reported complexes (Shen *et al.* 2011).

The hydrogen bonds lend stability to the structure. The packing plot of this compound is shown in Fig. 2. In the phenolate ligands, the proton of the phenolic hydroxy group transfers to the imine N atom. This proton is also involved in an intramolecular N—H···O hydrogen bond that imposes a nearly planar conformation on each ligand, with dihedral angles of 2.78 (3) and 2.43 (5)° between the aromatic rings of each ligand. In the crystal structure, molecules are linked by intermolecular O—H···O hydrogen bonds.

### Experimental

Reagents and solvents used were of commercially available quality. The Schiff base ligand 2-methoxy-6-(*N*-phenylcarboximidoyl)phenol was synthesized from condensation of *o*-vanillin and aniline. 1 mmol H*L*ligand was dissolved in ethanol(20 ml), then 0.5 mmol Manganese nitrate solution (in ethanol). The mixture solution was stirred for 4 h at room temperature. The resulting solid was filtered out and the solution evaporated yielding red crystals of compound (1) after several days.

### Refinement

The structure was solved by direct methods and successive Fourier difference synthesis. The H atoms bonded to C and N atoms were positioned geometrically and refined using a riding model [aliphatic C—H =0.96 Å (*U*_iso_(H) = 1.2*U*_eq_(C)), aromatic C—H = 0.93 Å (*U*_iso_(H) = 1.2 *U*_eq_(C)) and N—H = 0.86 Å with *U*_iso_(H) = 1.2*U*_eq_ (N)]. Water H atoms bonded to O atoms were located in difference Fourier maps and refined with O—H distance restraints of 0.83 (2)Å and *U*_iso_(H) = 1.5*U*_eq_(O).

### Crystal data

**Table:**

[Mn(C_14_H_13_NO_2_)_2_(H_2_O)_3_](NO_3_)_2_	*F*(000) = 1428
*M**_r_* = 687.52	*D*_x_ = 1.487 Mg m^−^^3^
Orthorhombic, *P**b**c**n*	Mo *K*α radiation, λ = 0.71073 Å
Hall symbol: -P 2n 2ab	Cell parameters from 2780 reflections
*a* = 17.4364 (3) Å	θ = 2.3–25.0°
*b* = 10.4199 (2) Å	µ = 0.50 mm^−^^1^
*c* = 16.9014 (3) Å	*T* = 296 K
*V* = 3070.74 (10) Å^3^	Block, red
*Z* = 4	0.26 × 0.14 × 0.06 mm

### Data collection

**Table:**

Bruker APEXII area-detector diffractometer	2705 independent reflections
Radiation source: fine-focus sealed tube	1864 reflections with *I* > 2σ(*I*)
graphite	*R*_int_ = 0.035
φ and ω scans	θ_max_ = 25.0°, θ_min_ = 2.3°
Absorption correction: multi-scan (*SADABS*; Sheldrick, 1996)	*h* = −18→20
*T*_min_ = 0.918, *T*_max_ = 0.969	*k* = −12→12
11534 measured reflections	*l* = −19→20

### Refinement

**Table:**

Refinement on *F*^2^	Primary atom site location: structure-invariant direct methods
Least-squares matrix: full	Secondary atom site location: difference Fourier map
*R*[*F*^2^ > 2σ(*F*^2^)] = 0.043	Hydrogen site location: inferred from neighbouring sites
*wR*(*F*^2^) = 0.118	H-atom parameters constrained
*S* = 1.07	*w* = 1/[σ^2^(*F*_o_^2^) + (0.0599*P*)^2^] where *P* = (*F*_o_^2^ + 2*F*_c_^2^)/3
2705 reflections	(Δ/σ)_max_ < 0.001
210 parameters	Δρ_max_ = 0.22 e Å^−^^3^
4 restraints	Δρ_min_ = −0.33 e Å^−^^3^

### Special details

**Table:**

Geometry. All e.s.d.'s (except the e.s.d. in the dihedral angle between two l.s. planes) are estimated using the full covariance matrix. The cell e.s.d.'s are taken into account individually in the estimation of e.s.d.'s in distances, angles and torsion angles; correlations between e.s.d.'s in cell parameters are only used when they are defined by crystal symmetry. An approximate (isotropic) treatment of cell e.s.d.'s is used for estimating e.s.d.'s involving l.s. planes.
Refinement. Refinement of *F*^2^ against ALL reflections. The weighted *R*-factor *wR* and goodness of fit *S* are based on *F*^2^, conventional *R*-factors *R* are based on *F*, with *F* set to zero for negative *F*^2^. The threshold expression of *F*^2^ > σ(*F*^2^) is used only for calculating *R*-factors(gt) *etc*. and is not relevant to the choice of reflections for refinement. *R*-factors based on *F*^2^ are statistically about twice as large as those based on *F*, and *R*- factors based on ALL data will be even larger.

### Fractional atomic coordinates and isotropic or equivalent isotropic displacement parameters (Å^2^)

**Table:**

	*x*	*y*	*z*	*U*_iso_*/*U*_eq_	
Mn1	0.5000	0.33828 (5)	0.2500	0.0421 (2)	
O1	0.38704 (9)	0.30891 (16)	0.29244 (10)	0.0460 (5)	
O1W	0.46876 (11)	0.3817 (2)	0.12448 (11)	0.0662 (6)	
H1WA	0.4674	0.3323	0.0845	0.099*	
H1WB	0.4722	0.4555	0.1034	0.099*	
O2	0.43669 (10)	0.13545 (17)	0.19361 (11)	0.0597 (6)	
O2W	0.5000	0.5493 (2)	0.2500	0.0604 (8)	
H2WA	0.5076	0.6037	0.2134	0.091*	
N1	0.27417 (11)	0.4077 (2)	0.37372 (11)	0.0414 (5)	
H1A	0.3225	0.4015	0.3645	0.050*	
C1	0.15746 (18)	0.6407 (3)	0.48555 (17)	0.0601 (8)	
H1	0.1066	0.6663	0.4902	0.072*	
C2	0.2127 (2)	0.7023 (3)	0.52808 (17)	0.0611 (8)	
H2	0.1996	0.7697	0.5615	0.073*	
C3	0.28780 (19)	0.6644 (3)	0.52134 (17)	0.0606 (8)	
H3	0.3255	0.7060	0.5507	0.073*	
C4	0.30785 (16)	0.5658 (3)	0.47178 (15)	0.0518 (7)	
H4	0.3588	0.5402	0.4678	0.062*	
C5	0.25210 (14)	0.5048 (2)	0.42797 (14)	0.0404 (6)	
C6	0.17617 (15)	0.5410 (3)	0.43588 (16)	0.0532 (7)	
H6	0.1381	0.4982	0.4078	0.064*	
C7	0.23079 (14)	0.3276 (2)	0.33641 (15)	0.0418 (6)	
H7	0.1785	0.3294	0.3470	0.050*	
C8	0.25749 (14)	0.2381 (2)	0.28081 (14)	0.0395 (6)	
C9	0.33671 (14)	0.2317 (2)	0.26127 (14)	0.0394 (6)	
C10	0.35947 (15)	0.1353 (2)	0.20736 (15)	0.0436 (6)	
C11	0.30689 (17)	0.0538 (2)	0.17469 (16)	0.0536 (7)	
H11	0.3233	−0.0087	0.1393	0.064*	
C12	0.22898 (16)	0.0622 (3)	0.19330 (16)	0.0533 (7)	
H12	0.1941	0.0060	0.1703	0.064*	
C13	0.20462 (16)	0.1525 (3)	0.24499 (15)	0.0479 (7)	
H13	0.1527	0.1585	0.2572	0.057*	
C14	0.46842 (18)	0.0327 (3)	0.1474 (2)	0.0848 (11)	
H14A	0.5233	0.0405	0.1460	0.102*	
H14B	0.4485	0.0369	0.0945	0.102*	
H14C	0.4547	−0.0481	0.1708	0.102*	
O5	0.03646 (13)	0.2937 (2)	0.48143 (13)	0.0812 (7)	
O3	0.04351 (13)	0.2360 (2)	0.36037 (13)	0.0807 (7)	
O4	−0.02028 (16)	0.1259 (2)	0.44494 (15)	0.0969 (8)	
N3	0.02000 (14)	0.2189 (3)	0.42868 (17)	0.0580 (6)	

### Atomic displacement parameters (Å^2^)

**Table:**

	*U*^11^	*U*^22^	*U*^33^	*U*^12^	*U*^13^	*U*^23^
Mn1	0.0316 (4)	0.0502 (4)	0.0447 (4)	0.000	0.0042 (2)	0.000
O1	0.0337 (10)	0.0565 (11)	0.0479 (11)	−0.0064 (9)	0.0031 (9)	−0.0088 (9)
O1W	0.0830 (14)	0.0674 (13)	0.0484 (11)	0.0038 (12)	−0.0137 (11)	−0.0008 (11)
O2	0.0445 (12)	0.0622 (12)	0.0725 (13)	0.0052 (9)	0.0152 (10)	−0.0130 (11)
O2W	0.080 (2)	0.0509 (17)	0.0506 (16)	0.000	0.0063 (14)	0.000
N1	0.0292 (11)	0.0542 (13)	0.0408 (12)	0.0027 (10)	0.0044 (10)	0.0003 (11)
C1	0.057 (2)	0.0640 (19)	0.0593 (19)	0.0149 (16)	0.0125 (16)	−0.0020 (17)
C2	0.086 (3)	0.0493 (17)	0.0481 (18)	0.0032 (17)	0.0109 (17)	−0.0025 (15)
C3	0.072 (2)	0.0544 (17)	0.0554 (19)	−0.0096 (16)	−0.0029 (16)	−0.0059 (16)
C4	0.0437 (17)	0.0572 (17)	0.0545 (17)	−0.0043 (14)	−0.0016 (14)	−0.0017 (15)
C5	0.0382 (15)	0.0468 (14)	0.0363 (14)	0.0017 (12)	0.0056 (12)	0.0064 (13)
C6	0.0425 (17)	0.0627 (18)	0.0545 (16)	0.0024 (14)	−0.0021 (14)	−0.0039 (16)
C7	0.0298 (14)	0.0500 (14)	0.0458 (14)	−0.0028 (12)	0.0012 (11)	0.0035 (11)
C8	0.0338 (15)	0.0449 (14)	0.0397 (13)	−0.0032 (11)	−0.0004 (11)	0.0058 (11)
C9	0.0375 (15)	0.0440 (15)	0.0367 (14)	−0.0011 (13)	0.0002 (12)	0.0054 (12)
C10	0.0432 (17)	0.0434 (15)	0.0441 (16)	0.0031 (13)	0.0052 (13)	0.0026 (13)
C11	0.070 (2)	0.0427 (16)	0.0477 (17)	−0.0044 (15)	0.0059 (15)	−0.0051 (14)
C12	0.0534 (19)	0.0541 (17)	0.0525 (17)	−0.0150 (14)	−0.0026 (15)	0.0015 (16)
C13	0.0366 (16)	0.0565 (17)	0.0505 (17)	−0.0084 (13)	0.0004 (12)	0.0025 (15)
C14	0.072 (2)	0.082 (2)	0.101 (3)	0.014 (2)	0.027 (2)	−0.027 (2)
O5	0.0847 (17)	0.0844 (16)	0.0745 (15)	0.0036 (13)	−0.0044 (13)	−0.0299 (14)
O3	0.0851 (17)	0.0974 (18)	0.0595 (13)	−0.0022 (14)	0.0156 (13)	−0.0015 (14)
O4	0.128 (2)	0.0688 (15)	0.0942 (19)	−0.0224 (15)	0.0255 (17)	−0.0070 (15)
N3	0.0543 (16)	0.0531 (16)	0.0666 (17)	0.0138 (13)	0.0033 (14)	−0.0050 (17)

### Geometric parameters (Å, °)

**Table:**

Mn1—O1	2.1184 (16)	C3—H3	0.9300
Mn1—O1^i^	2.1184 (16)	C4—C5	1.377 (3)
Mn1—O2W	2.199 (3)	C4—H4	0.9300
Mn1—O1W	2.2365 (18)	C5—C6	1.383 (3)
Mn1—O1W^i^	2.2366 (18)	C6—H6	0.9300
Mn1—O2	2.5678 (19)	C7—C8	1.403 (3)
Mn1—O2^i^	2.5678 (19)	C7—H7	0.9300
O1—C9	1.302 (3)	C8—C13	1.419 (3)
O1W—H1WA	0.8500	C8—C9	1.422 (3)
O1W—H1WB	0.8501	C9—C10	1.413 (3)
O2—C10	1.366 (3)	C10—C11	1.366 (3)
O2—C14	1.436 (3)	C11—C12	1.397 (4)
O2W—H2WA	0.8500	C11—H11	0.9300
N1—C7	1.291 (3)	C12—C13	1.352 (4)
N1—C5	1.419 (3)	C12—H12	0.9300
N1—H1A	0.8600	C13—H13	0.9300
C1—C2	1.363 (4)	C14—H14A	0.9600
C1—C6	1.375 (3)	C14—H14B	0.9600
C1—H1	0.9300	C14—H14C	0.9600
C2—C3	1.372 (4)	O5—N3	1.218 (3)
C2—H2	0.9300	O3—N3	1.238 (3)
C3—C4	1.371 (4)	O4—N3	1.228 (3)
			
O1—Mn1—O1^i^	163.38 (9)	C2—C3—H3	119.7
O1—Mn1—O2W	98.31 (5)	C3—C4—C5	119.6 (3)
O1^i^—Mn1—O2W	98.31 (5)	C3—C4—H4	120.2
O1—Mn1—O1W	97.12 (7)	C5—C4—H4	120.2
O1^i^—Mn1—O1W	86.24 (7)	C4—C5—C6	119.9 (3)
O2W—Mn1—O1W	78.33 (5)	C4—C5—N1	119.0 (2)
O1—Mn1—O1W^i^	86.24 (7)	C6—C5—N1	121.1 (2)
O1^i^—Mn1—O1W^i^	97.12 (7)	C1—C6—C5	119.5 (3)
O2W—Mn1—O1W^i^	78.33 (5)	C1—C6—H6	120.3
O1W—Mn1—O1W^i^	156.67 (11)	C5—C6—H6	120.3
O1—Mn1—O2	66.86 (6)	N1—C7—C8	124.2 (2)
O1^i^—Mn1—O2	98.93 (6)	N1—C7—H7	117.9
O2W—Mn1—O2	145.39 (4)	C8—C7—H7	117.9
O1W—Mn1—O2	73.12 (7)	C7—C8—C13	119.2 (2)
O1W^i^—Mn1—O2	128.55 (7)	C7—C8—C9	120.6 (2)
O1—Mn1—O2^i^	98.93 (6)	C13—C8—C9	120.2 (2)
O1^i^—Mn1—O2^i^	66.86 (6)	O1—C9—C10	120.7 (2)
O2W—Mn1—O2^i^	145.39 (4)	O1—C9—C8	122.1 (2)
O1W—Mn1—O2^i^	128.55 (7)	C10—C9—C8	117.1 (2)
O1W^i^—Mn1—O2^i^	73.12 (7)	C11—C10—O2	126.4 (2)
O2—Mn1—O2^i^	69.21 (8)	C11—C10—C9	120.9 (2)
C9—O1—Mn1	125.39 (15)	O2—C10—C9	112.7 (2)
Mn1—O1W—H1WA	129.7	C10—C11—C12	121.5 (2)
Mn1—O1W—H1WB	124.3	C10—C11—H11	119.3
H1WA—O1W—H1WB	102.5	C12—C11—H11	119.3
C10—O2—C14	118.1 (2)	C13—C12—C11	119.6 (3)
C10—O2—Mn1	111.19 (15)	C13—C12—H12	120.2
C14—O2—Mn1	130.53 (16)	C11—C12—H12	120.2
Mn1—O2W—H2WA	131.8	C12—C13—C8	120.6 (3)
C7—N1—C5	128.2 (2)	C12—C13—H13	119.7
C7—N1—H1A	116.0	C8—C13—H13	119.7
C5—N1—H1A	115.9	O2—C14—H14A	109.5
C2—C1—C6	120.7 (3)	O2—C14—H14B	109.5
C2—C1—H1	119.7	H14A—C14—H14B	109.5
C6—C1—H1	119.7	O2—C14—H14C	109.5
C1—C2—C3	119.7 (3)	H14A—C14—H14C	109.5
C1—C2—H2	120.2	H14B—C14—H14C	109.5
C3—C2—H2	120.2	O5—N3—O4	118.4 (3)
C4—C3—C2	120.6 (3)	O5—N3—O3	120.8 (3)
C4—C3—H3	119.7	O4—N3—O3	120.8 (3)

Symmetry codes: (i) −*x*+1, *y*, −*z*+1/2.

### Hydrogen-bond geometry (Å, °)

**Table:**

*D*—H···*A*	*D*—H	H···*A*	*D*···*A*	*D*—H···*A*
O1W—H1WA···O5^ii^	0.85	2.18	3.032 (3)	180
O1W—H1WB···O4^iii^	0.85	1.96	2.809 (3)	180
O2W—H2WA···O3^iii^	0.85	1.96	2.800 (3)	169
N1—H1A···O1	0.86	1.92	2.611 (2)	137

Symmetry codes: (ii) −*x*+1/2, −*y*+1/2, *z*−1/2; (iii) *x*+1/2, *y*+1/2, −*z*+1/2.
